# The E3 ligase ABI3-INTERACTING PROTEIN2 negatively regulates FUSCA3 and plays a role in cotyledon development in *Arabidopsis thaliana*

**DOI:** 10.1093/jxb/erx046

**Published:** 2017-03-28

**Authors:** Simon Duong, Eliana Vonapartis, Cheuk-Yan Li, Sajedabanu Patel, Sonia Gazzarrini

**Affiliations:** 1 Department of Biological Sciences, University of Toronto Scarborough, Toronto M1C 1A4, Canada; 2 Department of Cell and Systems Biology, University of Toronto, Toronto M5S 3G5, Canada

**Keywords:** AIP2, E3 ligase, embryogenesis, FUSCA3, post-translational regulation, protein degradation, protein localization, protein–protein interaction, seed development, transcription factor.

## Abstract

FUSCA3 (FUS3) is a short-lived B3-domain transcription factor that regulates seed development and phase transitions in *Arabidopsis thaliana*. The mechanisms controlling FUS3 levels are currently poorly understood. Here we show that FUS3 interacts with the RING E3 ligase ABI3-INTERACTING PROTEIN2 (AIP2). AIP2–green fluorescent protein (GFP) is preferentially expressed in the protoderm during early embryogenesis, similarly to FUS3, suggesting that their interaction is biologically relevant. FUS3 degradation is delayed in the *aip2-1* mutant and FUS3–GFP fluorescence is increased in *aip2-1*, but only during mid-embryogenesis, suggesting that FUS3 is negatively regulated by AIP2 at a specific time during embryogenesis. *aip2-1* shows delayed flowering and therefore also functions post-embryonically to regulate developmental phase transitions. Plants overexpressing *FUS3* post-embryonically in the L1 layer (*ML1p:FUS3*) show late flowering and other developmental phenotypes that can be rescued by *ML1p:AIP2*, further supporting a negative role for AIP2 in FUS3 accumulation. However, additional factors regulate FUS3 levels during embryogenesis, as *ML1:AIP2* seeds do not resemble *fus3-3*. Lastly, targeted expression of a RING-inactive AIP2 variant to the protoderm/L1 layer causes FUS3 and ABI3 overexpression phenotypes and defects in cotyledon development. Taken together, these results indicate that AIP2 targets FUS3 for degradation and plays a role in cotyledon development and flowering time in Arabidopsis.

## Highlight

FUSCA3 is revealed to be a new substrate for the E3 ligase ABI3-INTERACTING PROTEIN2 (AIP2), a negative regulator of ABI3. AIP2 targets FUSCA3 for degradation and regulates cotyledon development and flowering time.

## Introduction

Plants are sessile organisms that rely on hormones to regulate various aspects of their growth and development under different environmental stimuli. Plant development can be divided into three major phases: embryonic, vegetative, and reproductive development. The transitions between these phases of development are crucial for normal growth and are characterized by spatiotemporal expression of phase-specific genes that determine how the transitions between phases occur. One important developmental transition is that from embryogenesis to germination. During seed development the embryo accumulates seed storage compounds, acquires desiccation tolerance, and remains dormant until suitable environmental cues trigger germination. The *ABSCISIC ACID INSENSITIVE3* (*ABI3*), *FUSCA3* (*FUS3*), and the *LEAFY COTYLEDON* (*LEC*) genes play important and complementary role in the regulation of these processes in *Arabidopsis thaliana* (Finkelstein *et al.*, 2008; Holdsworth *et al.*, 2008; Santos-Mendoza *et al.*, 2008; Suzuki and McCarty, 2008; Nambara *et al.*, 2010; Finkelstein 2013). In particular, *abi3* and *fus3* mutant alleles have reduced accumulation of seed storage compounds, exhibit precocious germination of green, immature seeds, and fail to establish desiccation tolerance (Koorneef *et al.*, 1984; Nambara *et al.*, 1992, 1995; Keith *et al.*, 1994; Meinke *et al.*, 1994; Parcy *et al.*, 1994; Nambara *et al.*, 1995; Raz *et al.*, 2001). Loss-of-function and overexpression studies have shown that ABI3 and FUS3 act synergistically to regulate late embryonic development and inhibit vegetative growth (Keith *et al.*, 1994; Parcy *et al.*, 1994, 1997; Kroj *et al.*, 2003; Gazzarrini *et al.*, 2004; To *et al.*, 2006; Roscoe *et al.*, 2015). This agrees with the fact that both genes encode proteins of the B3-domain family of transcription factors (Giraudat *et al.*, 1992; Luerssen *et al*, 1998) that bind to the conserved RY motif in the promoter of target genes (Reidt *et al.*, 2000; Mönke *et al.*, 2004).

ABI3 and FUS3 play important roles in hormone signaling and synthesis, respectively. ABI3 is a positive regulator of ABA signaling, and its expression is induced by abscisic acid (ABA). FUS3 promotes ABA synthesis, and elevated ABA levels promote FUS3 protein stability (Koornneef, 1984; Nambara *et al.*, 1992, 2000; Lopez-Molina *et al.*, 2002; Gazzarrini *et al.*, 2004; Zhang *et al.*, 2005). FUS3 represses gibberellic acid (GA) synthesis and ethylene signaling, both of which promote germination, and in turn GA reduces FUS3 protein accumulation during vegetative development (Curaba *et al.*, 2004; Gazzarrini *et al.*, 2004; Lumba *et al.*, 2012). Finally, the expression of both genes is induced by auxin in the root, and ABI3/VP1 have been shown to regulate lateral root development positively and inhibit germination in response to auxin (Suzuki *et al.*, 2001; Brady *et al.*, 2003; Gazzarrini *et al.*, 2004; Liu *et al.*, 2013). This suggests a complex interaction between these master regulators of late embryogenesis and the hormone network, with positive and negative regulatory feedback loops.

Regulated proteolysis through the ubiquitin–proteasome system (UPS) plays important roles in all organisms studied to date, and ~5% of the Arabidopsis genome encodes components of this pathway (Smalle and Vierstra, 2004). Despite the important roles played by the UPS in several developmental processes, only a few substrates have been identified for the >1000 E3 ligases present in the Arabidopsis genome (Moon *et al.*, 2004; Vierstra, 2009; Kelley and Estelle, 2012; Lyzenga and Stone, 2012; Chen and Hellmann, 2013; Stone, 2014). The UPS plays a major role in the regulation of ABA biosynthesis and signaling components, as well as abiotic stress responses (Lyzenga and Stone, 2012; Stone, 2014). ABI3 is degraded through the 26S proteasome pathways by ABI3-INTERACTING PROTEIN2 (AIP2), a RING-H2 E3 ligase (Zhang *et al.*, 2005). Consistent with ABI3’s positive role in ABA signaling, the *aip2-1* mutant is hypersensitive to ABA, while *AIP2* overexpression shows reduced ABA sensitivity during germination. *AIP2* and *ABI3* show some overlapping expression profiles, as both are expressed during embryogenesis (Giraudat *et al.*, 1992; Baumbusch *et al.*, 2004; Zhang *et al.*, 2005). *AIP2* is also expressed in vegetative and reproductive tissues, suggesting it may have other roles during post-embryonic development (Zhang *et al.*, 2005). Regulation of ABI3 by ubiquitination has also been shown in rice. OsABI3 interacts with and is ubiquitinated by the rice ortholog of AIP2, DELAYED SEED GERMINATION1 (OsDSG1). Reduction of *DSG1* levels results in delayed germination and high tolerance to salt and drought stresses, while *DSG1* overexpression reduces plant stature (Park *et al.*, 2010). Similarly, two wheat AIP2 isoforms interact with the B2 and B3 domains of TaVP1 and induce earlier flowering, promote germination, and reduce ABA sensitivity when overexpressed in the Arabidopsis *aip2-1* mutant (Gao *et al.*, 2014).

E3 ligases can have more than one target in a conserved gene family (Joazeiro and Weissman, 2000). This has been shown for several E3 ligases involved in hormone signaling. The F-Box SLEEPY1 targets multiple DELLA repressors of GA signaling through interaction with the conserved GRAS motif (Dill *et al.*, 2004; Fu *et al.*, 2004; Ariizumi *et al.*, 2011). Furthermore, ETHYLENE INSENSITIVE 3 (EIN3) Binding F-Box 1 (EBF1) and EBF2 target EIN3 and the related EIN3-like1 (EIL1) proteins for degradation (Guo and Ecker, 2003; Potushack *et al.*, 2003; An *et al.*, 2010). Lastly, the RING KEG degrades the bZIP transcription factors ABRE-binding factor1 (ABF1), ABF3, and ABI5 through the 26S proteasome (Stone *et al.*, 2006; Liu and Stone, 2010; Chen *et al.*, 2013). AIP2 has a stronger binding affinity for the conserved B2+B3 domains of Arabidopsis ABI3 and also binds to the B1+B2 domains of rice and wheat ABI3 (Kurup *et al.*, 2000; Zhang *et al.*, 2005; Park *et al.*, 2010; Gao *et al.*, 2014), suggesting that AIP2 may be involved in regulated proteolysis of other members of the B3 domain family of transcription factors.

FUS3 is rapidly degraded through the 26S proteasome, but the mechanisms regulating FUS3 levels are still unclear (Lu *et al.*, 2010). Due to the high sequence conservation of the B2 and B3 domains of ABI3 and FUS3, overlap in the temporal and spatial domains of expression of *ABI3* and *FUS3* during embryonic development, and similarities in *abi3* and *fus3* mutant phenotypes, we tested whether FUS3 could be a substrate of AIP2. In this study, we show that FUS3 and AIP2 interact in yeast two-hybrid (Y2H) assay, *in vitro*, and *in planta*, and that FUS3 is degraded by AIP2. However, genetic analysis and targeted AIP2 expression to the epidermis suggest that additional factors regulate FUS3 abundance during embryogenesis, and that AIP2 may have additional targets other than FUS3 and ABI3. Lastly, loss-of-function and overexpression studies suggest that AIP2 plays a role in flowering time and cotyledon development in Arabidopsis.

## Materials and methods

### Plant material, growth conditions, and seed germination

Arabidopsis seeds (Columbia ecotype) of various genotypes were germinated on half-strength Murashige and Skoog (MS) medium containing 5 mM MES (pH 5.7). For germination assays, 150 two-week-old seeds were surface sterilized, chilled for 1 d, and germinated for 7 d at 21 °C under constant light. Experiments were repeated three times. Seedlings transferred to soil were grown in controlled environmental chambers at 21 °C under long-day cycles.

### Yeast two-hybrid assays

The *FUS3* (At3g26790) N-terminal region (FUS3^N90^) and *AIP2* (At5g20910) were amplified by PCR from Col-0 silique and seed cDNAs. *AIP2*^*(C/S*)^ was generated using Quickchange site-directed mutagenesis (Agilent Technologies; http://www.agilent.com) using primers described in Zhang *et al.* (2005). *FUS3* and *AIP2* variants were expressed in *Saccharomyces cerevisiae* as LexA DNA-binding domain (DB) and GAL4 activation domain (AD) fusions using the yeast plasmid expression vectors pEG202 and pJG4-5, respectively (Clontech; https://www.clontech.com). Primers used for cloning are listed in Supplementary Table S1 at *JXB* online. All the cloned constructs were transformed into EGY48 (Clontech). Transformants were plated on dropout medium (–His, –Trp, –Ura). Interaction assays were done by streaking transformants on –His/–Trp/–Leu/–Ura plates supplemented with 2% galactose and 1% raffinose.

### In vitro *GST pull-down assays*

FLAG-AIP2-6×His was cloned into the pET28 vector by PCR (Supplementary Table S1) to generate FLAG-AIP2-6×His. The glutathione *S*-transferase (GST)–FUS3 fusion construct has been described previously (Lu *et al.*, 2010). GST-tagged proteins were purified using glutathione resin (Sigma) and FLAG-AIP2-6×His by Ni-NTA-agarose affinity column chromatography (Qiagen; www.qiagen.com) according to the manufacturer’s recommendations. Pull-down assays were performed as described in Zhang *et al.* (2005) with purified GST–FUS3 as the bait and FLAG-AIP2-6×His as the prey. The pulled-down proteins were resolved by 12% SDS–PAGE, and immunodetection was carried out using polyclonal donkey horseradish peroxidase (HRP)-conjugated anti-GST antibody (GE Healthcare, http://www.gehealthcare.com). The bound antibodies were detected by SuperSignal West Pico chemiluminescent substrate (https://www.thermofisher.com).

### Generation of transgenic plants

The *35Sp:HA-AIP2* and *35Sp:HA-AIP2*^*C/S,E/G*^ constructs were created by replacing green fluorescent protein (GFP) with AIP2-HA variants in pEGAD (Cutler *et al.*, 2000) generated by PCR. *AIP2*^*C/S,E/G*^ was generated by site-directed mutagenesis. The *ML1p:HA-AIP2* and *ML1p:HA-AIP2*^*C/S,E/G*^ constructs were created by replacing the 35S promoter of *35Sp:HA-AIP2* and *35Sp:HA-AIP2*^*C/S,E/G*^ constructs with a PCR-generated 3 kb *ML1* promoter. The transcriptional reporter *AIP2p:GFP* was created by replacing the *35S* promoter of *pEGAD* with a PCR-generated 2 kb *AIP2* promoter. The translational reporter *AIP2p:AIP2-GFP* was created by cloning the *AIP2* cDNA in the *AIP2p:GFP* construct. All constructs were transformed into Arabidopsis by the floral dip method (Clough and Bent, 1998). The transgenic lines were selected on 50 μg ml^–1^ glufosinate ammonium salt (BASTA, Crescent Chemical; http://creschem.com). *ML1p:HA-AIP2 ML1p:FUS3-GFP* double transgenic plants were generated by crossing the parent plants.

### Bimolecular fluorescent complementation (BiFC)

The *cYFP–AIP2* construct was made by cloning the *AIP2* CDS into the cYFP (C-terminal fragment of yellow fluorescent protein) vector via the Gateway system (Invitrogen). The donor vector of AIP2 (pDONR201) was obtained from ABRC. *nYFP–FUS3* and *nYFP–MYB49* have been previously described (Tsai and Gazzarrini, 2012*a*). The BiFC constructs were transformed into *Agrobacterium tumefaciens* strain GV3101. *Nicotiana benthamiana* plants were agroinfiltrated as previously described (Lewis *et al.*, 2008) and plants were imaged after 48 h by confocal microscopy as described (Lu *et al.*, 2010).

### Cell-free degradation assays

Cell-free degradation assays were conducted as previously described (Lu *et al.*, 2010). GST–FUS3 was detected with anti-GST polyclonal antibody. Band intensities on western blots were quantified using ImageJ (https://imagej.nih.gov/ij/index.html). Pixel values were measured on equal-sized areas. The intensity values shown herein are the ratios relative to the time 0 treatment. Averages of three biological replicates with SDs are shown.

### Immunoblot analysis

Flowers were crushed in liquid nitrogen and homogenized in extraction buffer containing 50 mM Tris–HCl (pH 8), 150 mM NaCl, 1 mM EDTA, 10% (v/v) glycerol, 1% Triton X-100, 1 mM phenylmethylsulphonyl fluoride (PMSF), and 1× plant protease inhibitor cocktail (Sigma-Aldrich; http://www.sigmaaldrich.com). The concentrations of the protein extracts were measured by Bradford assay using Quick Start Bradford dye reagent (Biorad; http://www.bio-rad.com). The HA-tagged fusion proteins were detected with anti-HA polyclonal antibody (Cedarlane; https://www.cedarlanelabs.com) and peroxidase-AffiniPure Donkey Anti-Rabbit secondary antibody (Jackson Immunoresearch; https://www.jacksonimmuno.com). SuperSignal West Pico chemiluminescent substrate was used for band detection.

### Confocal microscopy

Confocal microscopy was performed using a Zeiss LSM 510 META confocal microscope. GFP was excited at 488 nm and detected at 505–530 nm (green channel), and autofluorescence from chlorophyll was detected at ≥585 nm (red channel). All dissected embryos were mounted in water. GFP quantification was performed using ImageJ (https://imagej.nih.gov/ij/index.html). Three areas of the embryo (cotyledon, hypocotyl, and root meristem) were selected, and the average GFP intensities of 20 nuclei/area were calculated. Averages of three samples with SDs are shown.

### RT–PCR and quantitative PCR

Total RNA was extracted from seeds at 24 h after imbibition and quantitative reverse transcription–PCR (qRT–PCR) was conducted as previously described (Lu *et al.*, 2010). For RT–PCR, primers spanning exons 1 and 2 of *AIP2* are 5'-GCTGAGATTCGAAGCATCC-3' and 5'-GCTTAACTGCTCCTTAGCTTGAG-3'. Primers spanning exons 4 and 5 are 5'-GTTATTGGCGACAAGATGC-3' and 5'-GTACATATATTCACCTCCGCG-3'.

*ACTIN7* was used as the loading control.

## Results

### *AIP2 interacts with FUS3 in YTH assay*, in vitro, *and* in planta

**Fig. 1.**
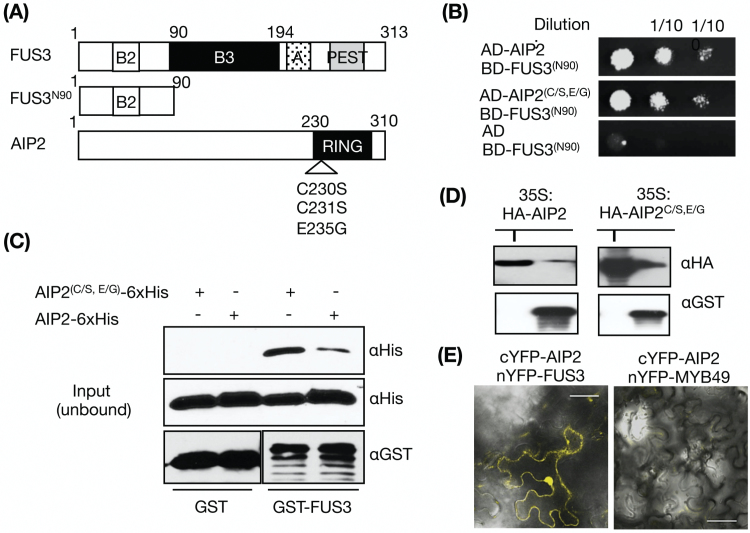
AIP2 interacts with FUS3 in yeast-two hybrid assay, *in vitro*, and *in planta*. (A) Schematic diagram of FUS3 and AIP2 variants used in Y2H and pull-down assays. The B2 and B3 basic domains, the activation (A) domain, and the PEST degradation motif of FUS3 are shown. The location of C230S, C231S, and E235G mutations in AIP2^(C/S, E/G)^ are indicated by the triangle. Numbers refer to the amino acid residues in the proteins. (B) Y2H assays showing interaction between BD-FUS3^(N90)^ and AD-AIP2 variants on selective media. The empty pJG4-5 vector containing the B42 activation domain (AD) was used as the negative control. BD, LexA DNA-binding domain. (C) *In vitro* pull-down assays of GST–FUS3 (~66 kDa) with AIP2-6×His and inactive AIP2^(C/S,E/G)^-6×His (~40 kDa). Immunoblots using anti-His and anti-GST antibodies show interaction of AIP2 and AIP2^(C/S, E/G)^ with FUS3. GST was used as the negative control. (D) *In vitro* pull-down assay showing interaction of FUS3 with AIP2 and its variants. GST–FUS3 (2.5 µg) was incubated with *35Sp:HA-AIP2* or *35Sp:HA-AIP2*^*C/S,E/G*^ plant cell lysates, pulled-down using glutathione resin, and detected with anti-GST antibody. The AIP2 and *AIP2*^*C/S,E/G*^ were detected with anti-HA antibody. I, input protein sample. (E) Confocal images showing interaction between FUS3 and AIP2 by BiFC in *N. benthamiana*. Transient co-expression of nYFP–FUS3 with cYFP–AIP2 showing YFP fluorescence in the nucleus and cytoplasm of pavement cells. No fluorescence was detected when the negative control, nYFP–MYB49, was co-expressed with cYFP–AIP2. The same confocal settings were used in both images. Scale bars=50 ìm. (This figure is available in colour at *JXB* online.)

To test whether AIP2 also interacts with the conserved B2 domain of FUS3, a deletion construct containing the first 90 amino acids of FUS3 (FUS3^N90^) including the B2 domain was generated (Fig. 1A). The full-length FUS3 protein could not be stably expressed in yeast and hence could not be used in Y2H assays (Tsai and Gazzarrini, 2012*a*). Co-expression of FUS3^N90^ and AIP2 could activate the leucine reporter (Fig. 1B), suggesting that the N-terminal region of FUS3 is sufficient for the interaction with AIP2 in yeast. To verify the interaction observed in yeast, *in vitro* GST pull-down assays were performed using recombinant GST–FUS3 as the bait and AIP2-His as the prey proteins. After incubation of both purified bait and prey proteins, FUS3 was proficient in pulling down AIP2 (Fig. 1C). FUS3 was also able to pull-down AIP2 from cell lysates of plants overexpressing AIP2 (35Sp:HA-AIP2; Fig. 1D). We then generated an inactive AIP2 variant (AIP2^C/S,E/G^) carrying two mutations (C230S and C231S) in the RING domain previously shown to inactivate the protein (Zhang *et al.*, 2005) and a third mutation in a well-conserved residue (E235G) (Fig. 1A). FUS3 also interacted with the RING-inactive AIP2^C/S,E/G^ variant in yeast and pull-down assays (Fig. 1B, C, D), in agreement with previous findings showing that mutations in the RING domain do not interfere with the ability of AIP2 to interact with ABI3 (Zhang *et al.*, 2005). Lastly, we tested whether AIP2 and FUS3 interacted *in planta* using BiFC. We generated fusions of the YFP domains to FUS3 and AIP2 and transiently co-expressed them in *N. benthamiana* leaves. YFP fluorescence was observed in the nuclei and cytoplasm of pavement cells, signifying an *in planta* interaction between AIP2 and FUS3 (Fig. 1E). The dual localization of this interaction is in agreement with earlier studies showing a nuclear and cytoplasmic localization of FUS3 *in vivo* and in *N. benthamiana*, and of AIP2 in transient *N. benthamiana* assays (Gazzarrini *et al.*, 2004; Tsai and Gazzarrini, 2012*a*; Zhang *et al.*, 2005). Together, the data suggest that AIP2 and FUS3 interact and that the interaction occurs in the nuclei and cytosol.

### AIP2 expression pattern during embryogenesis

**Fig. 2.**
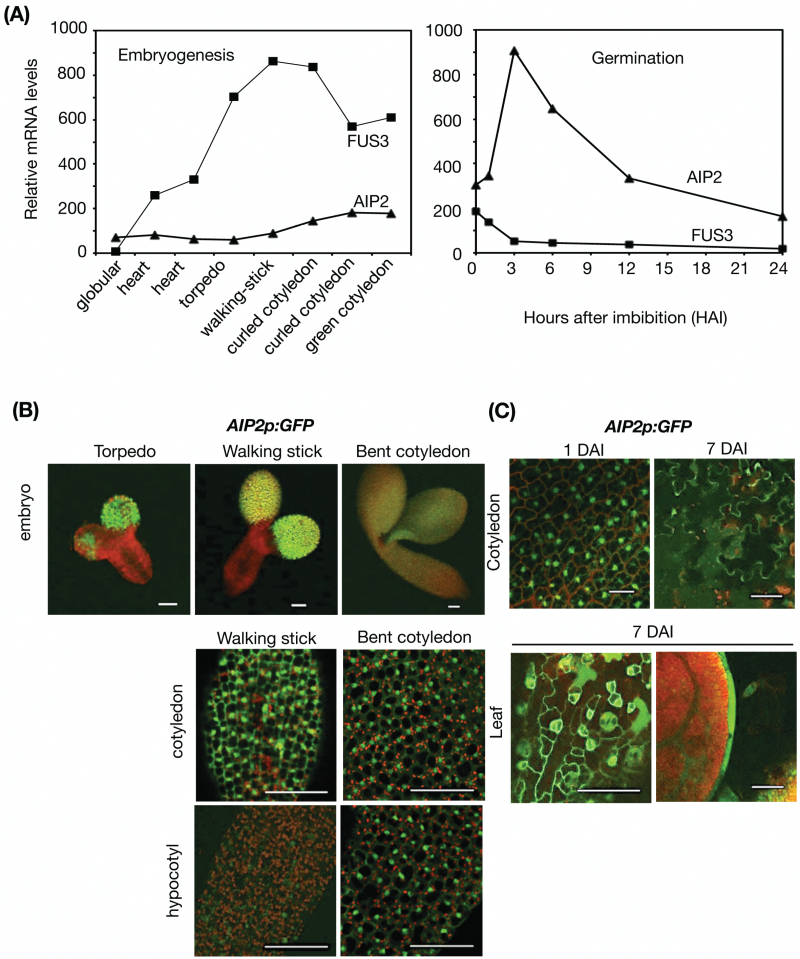
Expression pattern of AIP2 during embryogenesis and in seedlings. (A) *FUS3* and *AIP2* mRNA levels during embryogenesis (BAR; Toufighi *et al.*, 2005). (B) Confocal images of *AIP2p:GFP* embryos (top panels). Bottom panels show paradermal optical sections of walking-stick and bent-cotyledon embryos. (C) Confocal images of *AIP2p:GFP* seedlings. Top panels, paradermal optical sections of cotyledons; bottom panels, paradermal (left) and median longitudinal (right) optical sections of leaves of seedlings 7 days after imbibition (DAI). Scale bars=50 ìm. All images were taken under comparable confocal settings. (This figure is available in colour at *JXB* online.)

To test whether the interaction between these two proteins is biologically relevant, we examined whether the expression pattern of *AIP2* overlaps with that of *FUS3*. The temporal expression patterns of *AIP2* and *FUS3* were first analyzed during embryogenesis and germination using microarray data (Fig. 2A; BAR; Toufighi *et al.*, 2005). *FUS3* expression is greatest during mid-embryogenesis and decreases thereafter, reaching a very low level during germination. This is consistent with previous analyses of FUS3 transcript levels during embryogenesis and germination (Lu *et al.*, 2010). Interestingly, *AIP2* expression appears to be significantly lower than that of *FUS3* during embryogenesis. AIP2 transcript levels increase during late embryogenesis and its expression peaks during the first 3 h of seed imbibition, but decreases thereafter, showing an opposite trend compared with FUS3 transcripts.

**Fig. 3.**
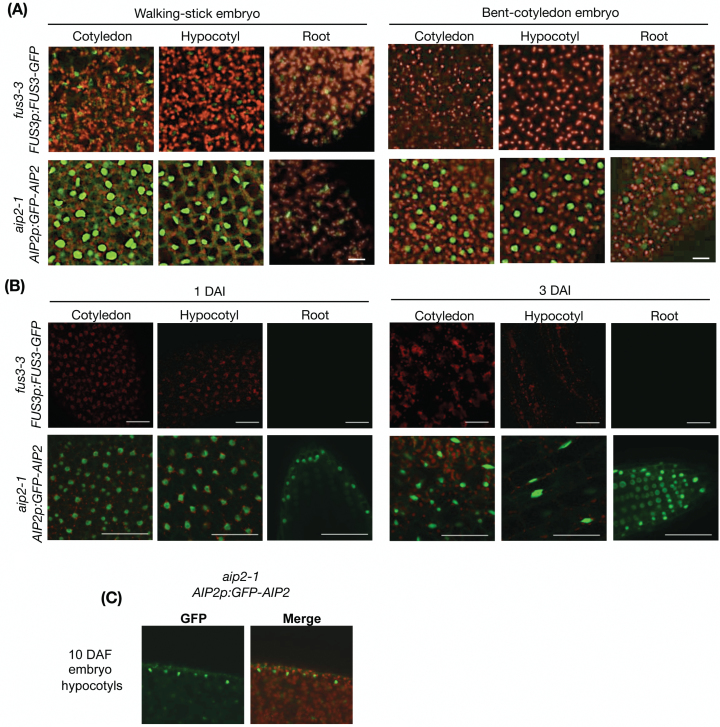
FUS3 and AIP2 protein localization patterns during embryogenesis and germination. (A) Confocal images showing GFP fluorescence in the protoderm of *fus3-3 FUS3p:FUS3-GFP* and *aip2-1 AIP2p:GFP-AIP2* embryos at walking-stick and bent-cotyledon stages of embryogenesis. (B) Confocal images of cotyledons, hypocotyls, and roots of *FUS3p:FUS3-GFP* and *AIP2p:GFP-AIP2* seedlings during germination. Longitudinal optical sections of the root and paradermal optical sections of the cotyledon and hypocotyl are shown. All images were taken under comparable confocal settings. Images shown were merged combining the GFP fluorescence and autofluorescence from chlorophyll. Scale bars=20 μm. (C) Median-longitudinal section of hypocotyls of an embryo 10 d after fertilization showing preferential localization of GFP–AIP2 to the protoderm. (This figure is available in colour at *JXB* online.)

Previously, an *AIP2p:GUS* reporter was used to show expression of *AIP2* in germinating seedlings (Zhang *et al.*, 2005). However, the spatial *AIP2* expression pattern during embryogenesis was not described. To determine the spatial location of *AIP2*, a transcriptional reporter was developed (*AIP2p:GFP*; Fig. 2B). According to the GFP localization pattern, the *AIP2* promoter is active during embryogenesis, in both cotyledons and hypocotyls of embryos. *AIP2* expression in the cotyledons is strong throughout embryogenesis, while that in hypocotyls gradually increases as the embryo progresses towards late embryogenesis. During post-embryonic development, *AIP2* is found in the epidermis of the cotyledons and young leaves (Fig. 2C). Similar expression patterns were found when using a translational reporter (*AIP2p:AIP2-GFP*) introduced into the *aip2-1* mutant, with AIP2–GFP showing both cytoplasmic and nuclear localization, while FUS3 showed cytoplasmic and nuclear localization at early embryo stages and was predominantly nuclear localized at later stages Figs 3A, B; Gazzarrini *et al.*, 2004). During embryogenesis, AIP2–GFP showed preferential localization to the protoderm (Fig 3C). We conclude that the spatial expression pattern of AIP2 during embryogenesis overlaps with that of FUS3 previously published, with both proteins showing preferential expression in the protoderm (Gazzarrini *et al.*, 2004). These data suggest that the interaction of AIP2 and FUS3 is biologically relevant and that their interaction *in vivo* is likely to occur in the protoderm of embryos.

### AIP2 negatively regulates FUS3

**Fig. 4.**
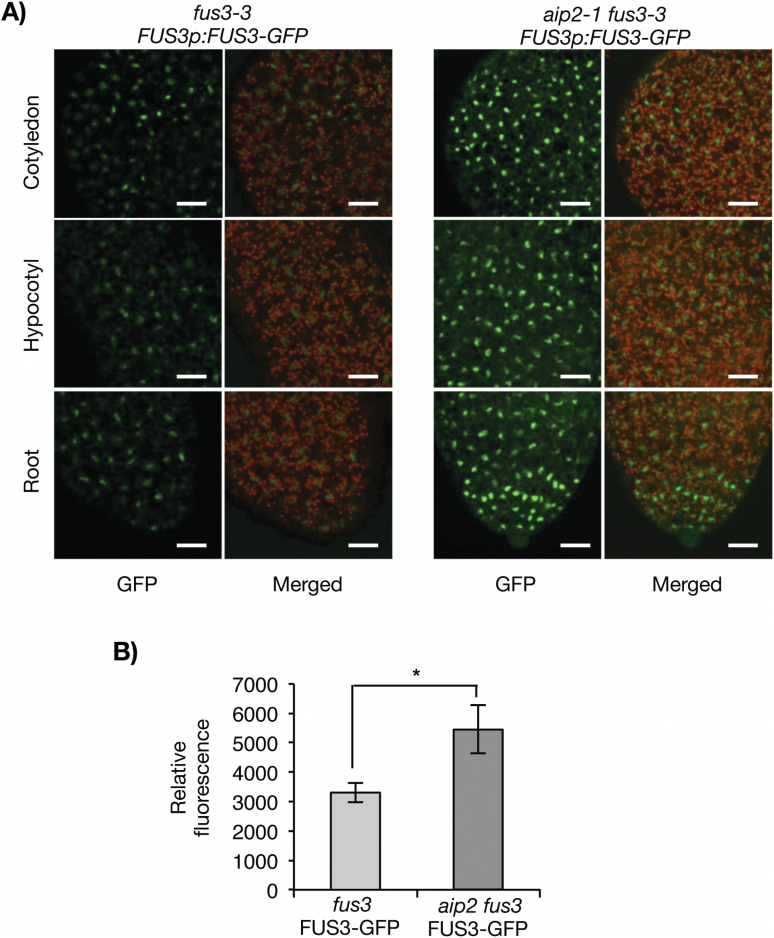
Lack of AIP2 increases the FUS3–GFP level during mid-embryogenesis. Confocal images (A) and quantification (B) showing increased FUS3–GFP fluorescence in *aip2-1 fus3-3 FUS3p:FUS3-GFP* compared with *fus3-3 FUS3p:FUS3-GFP* walking-stick embryos. All images were taken under comparable confocal settings. Images shown were merged combining the GFP fluorescence and autofluorescence from chlorophyll. Scale bar=20μm. (*) p < 0.05 (two-tailed t-test). (This figure is available in colour at *JXB* online.)

**Fig. 5.**
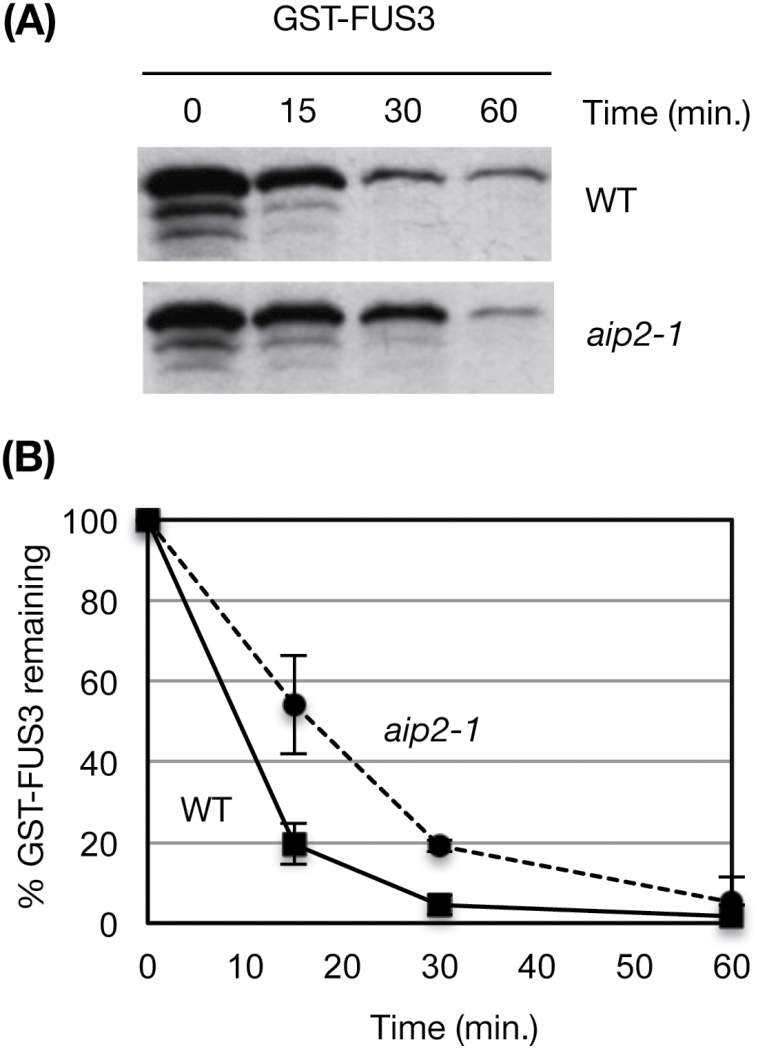
AIP2 negatively regulates FUS3. (A) Immunoblots of cell-free degradation assays showing glutathione *S*-transferase (GST)–FUS3 protein levels after incubation in wild-type (WT) and *aip2-1* lysates for 60 min. FUS3–GST was detected with anti-GST antibody. Protein lysates were extracted from seedlings at 7 days after imbibition (DAI). Three experiments were conducted and one representative is shown. (B) Degradation kinetics of GST–FUS3. Averages of three experiments ±SD are shown.

Previous studies using a *FUS3p:FUS3-GFP* translational reporter that rescues the *fus3-3* mutant have shown that FUS3 protein accumulates at a low level during embryogenesis, but is not detected in mature embryos or germinating seeds despite the presence of its mRNA (Figs 2, 3; Gazzarrini *et al.*, 2004; Lu *et al.*, 2010). In contrast, AIP2 is detected throughout embryogenesis, in mature embryos and germinating seeds (Fig. 3). To test whether AIP2 plays a role in degrading FUS3 during embryogenesis, we crossed the *FUS3p:FUS3-GFP* reporter line in the *aip2-1* mutant previously shown to lack *AIP2* transcripts and protein (Zhang *et al.* 2005). Interestingly, FUS3–GFP fluorescence was increased only during the walking-stick stage, suggesting that AIP2 negatively regulates FUS3 during mid-embryogenesis (Fig. 4; Supplementary Fig. S1). The effect of the *aip2-1* mutation on the level of FUS3 suggests that FUS3 interaction with and degradation by AIP2 appear to be spatially and temporally restricted to a specific developmental stage. Given that no AIP2 homologs are present that may cause a redundancy in activity with AIP2 (Zhang *et al.*, 2005), other E3 ligases or factors may maintain low levels of FUS3 throughout embryogenesis and especially during late embryogenesis (Lu *et al.*, 2010). To confirm further the role of AIP2 in FUS3 degradation, we used a cell-free degradation assay (Fig. 5). The degradation of FUS3 was delayed in the *aip2-1* mutant, indicating that AIP2 is a negative regulator of FUS3.

### *Overexpression of* AIP2 *rescues vegetative and reproductive phenotypes of plants overexpressing* FUS3

Overexpression of *FUS3* post-embryonically in the L1 layer (*ML1p:FUS3-GFP*) has a dramatic effect on the development of Arabidopsis plants, resulting in delayed embryonic-to-vegetative (germination) and vegetative-to-reproductive (flowering) phase transitions (Gazzarrini *et al.*, 2004; Tsai and Gazzarini, 2012*b*). Some of the *ML1p:FUS3-GFP* phenotypes include development of embryonic traits during vegetative growth, including cotyledon-like and glabrous leaves, stunted and bushy growth, and shorter and aborted siliques (Gazzarrini *et al.*, 2004).

**Fig. 6.**
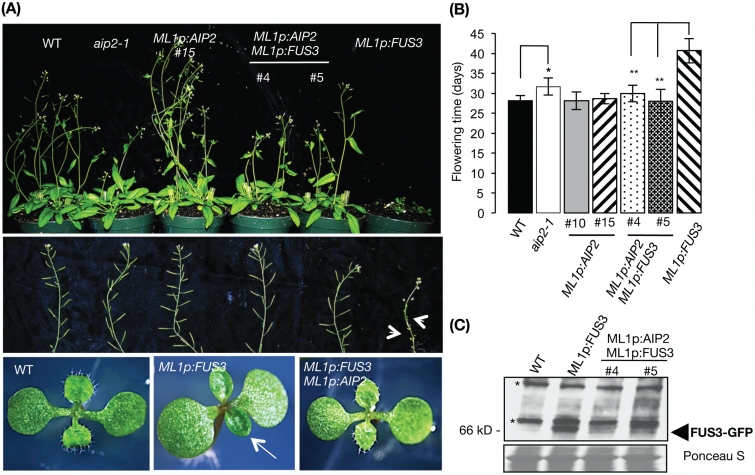
Rescue of *ML1p:FUS3* phenotypes by *ML1p:AIP2*. (A) *ML1p:FUS3-GFP* late flowering (top panel), aborted siliques (arrow heads; middle panel), and glabrous leaves (arrows; bottom panel) are rescued in *ML1p:FUS3-GFP ML1p:HA-AIP2* double transgenic plants. Plants were grown for 1 month on soil under a long-day growth cycle. (B) Quantification of flowering time (days to flowering) for genotypes shown in (A). *n*=10 plants for all genotypes. Only 5 out of 10 *ML1p:FUS3-GFP* plants flowered; *P<0.0002 from the wild type (WT); **P<0.02 from *ML1p:FUS3-GFP.* (C) Immunoblot of seedlings at 4 d after imbibition of two independent *ML1p:HA-AIP2 ML1p:FUS3-GFP* double transgenic plants. The blot was probed with GFP antibody to detect FUS3–GFP (~60 kDa). (*) cross-reacting bands. (This figure is available in colour at *JXB* online.)

To confirm further the role of AIP2 as a negative regulator of FUS3, we overexpressed *AIP2* under the *ML1* promoter (*ML1p:HA-AIP2*) in wild-type and *ML1p:FUS3-GFP* plants and selected transgenic lines that accumulated HA-AIP2 at different levels (Supplementary Figs S2A, S3A). Bolting assays show a dramatic rescue of the late flowering phenotype caused by FUS3 overexpression in *ML1p:FUS3-GFP ML1p:HA-AIP2* double transgenic plants (Fig. 6A, B). This is supported by a decrease in FUS3 protein levels in the double transgenic plants (Fig. 6C). The rescue was not due to transgene silencing, as *FUS3–GFP* mRNA was present in both rescued lines (Supplementary Fig. S3B). *ML1p:HA-AIP2 ML1p:FUS3-GFP* double transgenic plants also show a dramatic rescue of other *ML1p:FUS3-GFP* vegetative and reproductive phenotypes, in which the plants no longer exhibited cotyledon-like and glabrous leaves or aborted siliques (Fig. 6A). Interestingly, the *aip2-1* mutant shows a late flowering phenotype compared with the wild type; however, *ML1p:HA-AIP2* showed a similar flowering time to the wild type (Fig. 6B). Altogether, these data further support a role for AIP2 as a negative regulator of FUS3, and also indicate that AIP2 plays a role in flowering time.

### Role of AIP2 in cotyledon development

The *ML1* promoter is active in the protoderm throughout embryogenesis, and *ML1p:FUS3* can rescue the *fus3-3* mutant (Gazzarrini *et al.*, 2004). However, *ML1p:HA-AIP2* seeds were not desiccation intolerant like *abi3* or *fus3* mutant alleles, did not accumulate ectopic trichomes on cotyledons like the *fus3* mutant, and did not display an altered germination rate, suggesting that *ML1p:AIP2* is not able to degrade FUS3 fully during embryogenesis (Fig. 6; Supplementary Fig. S4). This is in agreement with similar findings in *35Sp:AIP2* lines (Zhang *et al.*, 2005) and suggests that other negative regulators or mechanisms control FUS3 and ABI3 levels during embryogenesis.

**Fig. 7.**
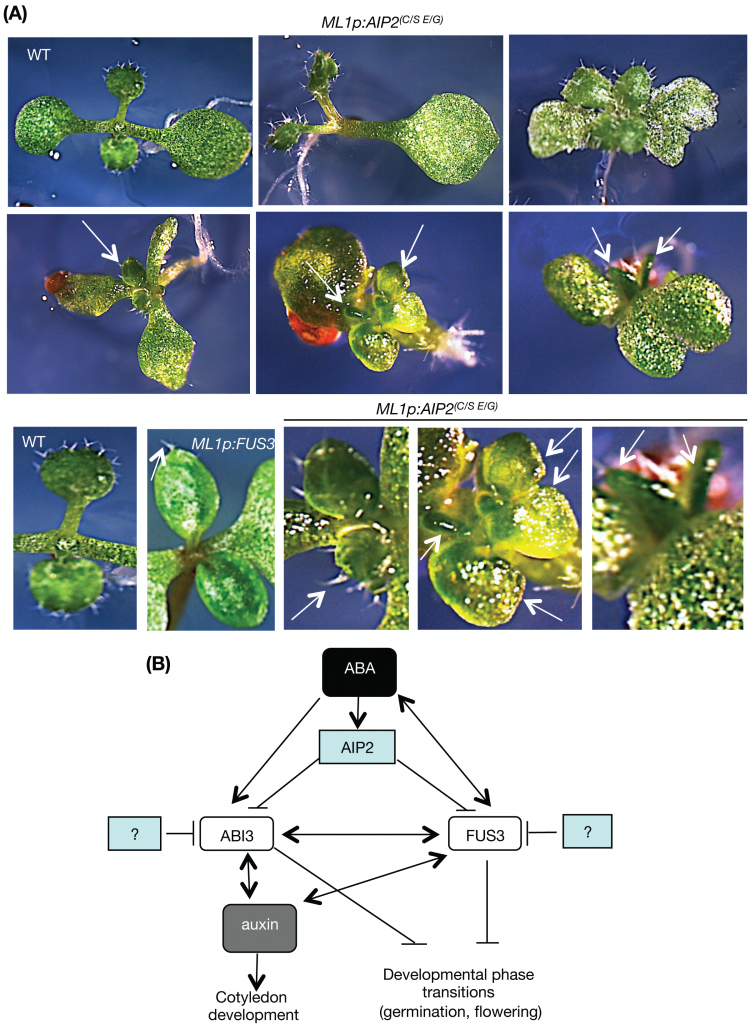
Phenotype of *ML1p:HA-AIP2*^(C/S,E/G)^ and model of the role of AIP2 in plant development. (A) Phenotypes of seedlings from two *ML1p:HA-AIP2*^(C/S,E/G)^ lines, showing defects in cotyledon development, including reduced or lack of chlorophyll, altered cotyledon number (monocotyledons and polycotyledons), and altered morphology (narrow cotyledons, cotyledon fusion). Arrows point to glabrous leaves or leaves showing few, unbranched trichomes, resembling *ML1p:FUS3* leaves, compared with the wild type (WT) (a close-up image of the WT is shown in the bottom, left corner panel). (B) Model showing the role of AIP2 as a negative regulator of FUS3 and ABI3. Other unknown negative regulators (such as E3s) or other mechanisms also control FUS3 and ABI3 protein levels. AIP2 may regulate cotyledon development through auxin. (This figure is available in colour at *JXB* online.)

To understand further the role of AIP2, we targeted the inactive *AIP2*^*C/S,E/G*^ variant to the L1 layer (Supplementary Fig. S2C). *ML1p:HA-AIP2*^*C/S,E/G*^ induced seedling growth arrest (Supplementary Table S2), glabrous leaves (Fig. 7A), and delayed growth and flowering (Supplementary Fig. S5). These phenotypes are also induced by *FUS3* overexpression (*ML1p:FUS3*; Figs 6A, 7A), and late flowering is shown in *35Sp:ABI3* plants, too (Zhang *et al.*, 2005). Interestingly, some *ML1p:HA-AIP2*^*C/S,E/G*^ lines caused a number of cotyledon defects visible at the seedling stage, including altered cotyledon number (mono- or polycotyledon), cotyledon fusions, cotyledon margin defects, and reduced or lack of chlorophyll (Fig. 7A). Tricotyledons appear in *fus3-3* at low frequency (Tsai and Gazzarrini, 2012*a*). However, neither *fus3-3* nor *abi3* mutants show the remaining cotyledon defects. Although the inactive *AIP2*^*(C/S,E/G*)^ lacks ubiquitination function, it can still interact with its substrates (Fig. 1; Zhang *et al.*, 2005). We conclude that *AIP2*^*(C/S,E/G*)^ may interfere with the regulation of AIP2 substrates—ABI3, FUS3, and other unknown targets—leading to their accumulation. Furthermore, changes in AIP2 function in the protoderm during embryogenesis and L1 layer during postembryonic development suggest a role for AIP2 in cotyledon development and flowering time.

## Discussion

Protein degradation is an important process by which cells regulate protein levels in response to a variety of signals. In plants, the rapid turnover of critical transcriptional activators or repressors is crucial for the regulation of developmental phase transitions as well as for the plant’s ability to respond to hormones and adapt to a changing environment (Stone and Callis, 2007; Vierstra, 2009; Kelley and Estelle, 2012; Stone, 2014). In Arabidopsis, the B3 domain transcription factors ABI3 and FUS3 are master regulators of embryonic functions, and interact at different levels to promote seed dormancy and inhibit germination (Santos-Mendoza *et al.*, 2008; Suzuki and McCarty, 2008; Nambara *et al.*, 2010; Finkelstein, 2013). Previous studies have shown that the RING-type E3 ubiquitin ligase, AIP2/DSG1, is a negative regulator of ABI3/VP1 in Arabidopsis and rice, and also interacts with ABI3/VP1 in wheat (Zhang *et al.*, 2005; Park *et al.*, 2010; Gao *et al.*, 2014). Here we demonstrate that in Arabidopsis AIP2 also interacts with and negatively regulates FUS3. FUS3 degradation is delayed in the *aip2-1* mutant, and a dramatic rescue of *FUS3* overexpression phenotypes is shown by *AIP2* overexpression. Although both FUS3 and AIP2 have overlapping expression patterns (protoderm of embryos) and subcellular localizations (nucleus and cytoplasm) during embryogenesis, lack of AIP2 increases FUS3 levels only during mid-embryogenesis, a time corresponding to the peak of ABA levels. Lastly, targeted overexpression of the RING-inactive *AIP2*^*C/S,E/G*^ to the protoderm and L1 layer suggests that AIP2 also plays a role in cotyledon development and flowering time. Collectively, these data indicate that AIP2 is a negative regulator of FUS3, and that AIP2 expression in the protoderm is important for proper cotyledon development during embryogenesis.

### AIP2 interacts with the B2 domain of FUS3

In this study we show that FUS3 and AIP2 interact in Y2H assay, *in vitro*, and *in planta*, and display predominantly nuclear but also cytoplasmic interaction. These findings are in agreement with previous subcellular localization of FUS3 in Arabidopsis and transient expression of AIP2 and FUS3 in *N. benthamiana* (Gazzarrini *et al.*, 2004; Zhang *et al.*, 2005; Tsai and Gazzarrini, 2012*a*; Gao *et al.*, 2014), as well as the *in vivo* AIP2 localization pattern shown in this study. Collectively, three independent methods indicate that AIP2 interacts with FUS3. Interestingly, the N-terminal domain of FUS3 containing the B2 domain is sufficient for the interaction with AIP2. Besides interacting with AIP2, the FUS3 N-terminus has been shown to interact with the SnRK1 kinase AKIN10, and to contain the site of FUS3 phosphorylation by AKIN10 within the B2 domain (Tsai and Gazzarrini, 2012*a*). Deletion of the B2 domain of FUS3, which also contains a putative nuclear localization signal (NLS), does not affect the nuclear localization of FUS3 (Lu *et al.*, 2010). Thus, the B2 domain of FUS3 is important for protein–protein interactions and phosphorylation, but not for nuclear localization.

The B2 domain of ABI3 is also important for protein–protein interaction. The B2+B3 domain of ABI3 binds to AIP2 in Arabidopsis, wheat, and oat, while the B1+B2 domain of ABI3 binds to DSG1/AIP2 in rice (Zhang *et al.*, 2005; Park *et al.*, 2010; Gao *et al.*, 2014). Furthermore, the B1+B2 domain of ABI3 is important for the interaction with an FRI-related protein in yellow cedar (Zeng *et al.*, 2013), a CONSTANS-related protein in Arabidopsis, as well as other proteins (Jones *et al.*, 2000; Kurup *et al.*, 2000). Although the B2 domain of ABI3 contains a putative NLS (Giraudat *et al.*, 1992), the B2 domain alone is not sufficient for ABI3 nuclear localization, but is required for transactivation (Marella and Quatrano, 2007). Thus, while the B3 domain of FUS3/ABI3/VP1-related proteins is required for DNA binding, the B1 and B2 domains may serve important regulatory functions, possibly through protein–protein interactions and protein modifications.

### AIP2 is expressed in the protoderm of embryos and negatively regulates FUS3

Previously, an *AIP2p:GUS* reporter was shown to be ubiquitously expressed in all tissues including mature embryos, vegetative tissues, and reproductive organs (Zhang *et al.*, 2005). We generated an *AIP2p:GFP* reporter and showed that the AIP2 promoter is strongly active in the protoderm of cotyledons at early embryonic stages and becomes active throughout the entire embryo at later stages. Using translational reporters, we show that both FUS3–GFP and AIP2–GFP fusion proteins are preferentially expressed in the protoderm of embryos, indicating that FUS3 and AIP2 expression and localization patterns partially overlap and the interaction is biologically relevant (Lu *et al.*, 2010).

Cell-free degradation assays show that FUS3 degradation is delayed in cell extracts of *aip2-1*. However, *in vivo* experiments show that FUS3–GFP levels are higher in the *aip2-1* mutant only during the walking-stick stage of embryogenesis, a time during which ABA levels are highest and AIP2 expression increases. This suggests that FUS3 degradation by AIP2 occurs at a specific developmental window and correlates with the highest ABA levels. Considering that *AIP2* expression is induced by ABA during vegetative growth (Zhang *et al.*, 2005), and that ABA stabilizes the FUS3 protein (Gazzarrini *et al.*, 2004), ABA may have a dual function: to increase FUS3 levels during mid-embryogenesis and at the same time to induce its cognate E3 ligase to reduce FUS3 accumulation once ABA levels decrease. This may prevent excessive FUS3 accumulation and maintain FUS3 homeostasis during mid-embryogenesis.

During late embryogenesis, when ABA levels drop, loss of AIP2 had no effect on the FUS3 protein (Gazzarrini *et al.*, 2004; Lu *et al.*, 2010). This suggests that other E3 ligases or other mechanisms prevent FUS3 protein accumulation at later embryonic stages, including lower ABA levels. Different E3 ligases can target the same substrate under different physiological conditions (Mazzucotelli *et al.*, 2006). This is true for ABA signaling components including ABI5, whose degradation is triggered by different E3 ligases and by different mechanisms. For example, ABI5 is degraded by the CUL4-based RING E3 ligases DWD hypersensitive to ABA 1 (DWA1), DWA2, and ABA-hypersensitive DCAF1 (ABD1), which promote ABI5 degradation in the presence of ABA (Lee *et al.*, 2010; Seo *et al.*, 2014). ABI5 is also degraded by the RING E3 ligase, KEEP-ON-GOING (KEG), which degrades ABI5 in the absence of stress. In the presence of stress or ABA, ABI5 accumulates through ABA-dependent KEG self-ubiquitination and proteasomal degradation (Stone *et al.*, 2006; Liu and Stone, 2010, 2013). The identification of additional negative regulators of FUS3 and ABI3 is required to understand fully the regulation of their levels and activities during embryogenesis.

AIP2 negatively regulates two major embryonic regulators, ABI3 and FUS3; however, plants with constitutive (*35Sp:AIP2*) or targeted (*ML1p:AIP2*) expression of *AIP2* do not produce seeds that resemble *fus3* or *abi3* mutant alleles. Several reasons may explain the lack of these phenotypes. First, FUS3 and ABI3 may be degraded by other E3s, as discussed above. Secondly, ABA is a positive regulator of FUS3 and ABI3, thus a high ABA level during embryogenesis may protect FUS3 and ABI3 from complete degradation by AIP2. Thirdly, while the 35S promoter is a strong promoter, *35Sp:FUS3* does not rescue the *fus3-3* mutant (Gazzarrini *et al.*, 2004), suggesting that *35Sp:AIP2* may not be expressed at the right time or place to degrade FUS3 fully during embryogenesis. Furthermore, although *ML1p:FUS3* rescues *fus3-3* (Gazzarrini *et al.*, 2004), *ML1p:AIP2* may not drive sufficient expression to degrade endogenous FUS3 and ABI3 fully. In support of these observations, post-embryonic overexpression of *AIP2* in plants overexpressing *FUS3* under the same promoter (*ML1p:HA-AIP2 ML1p:FUS3-GFP*) completely rescued the morphology designated to *FUS3*-overexpressing plants. Similarly to FUS3, *35Sp:ABI3* plants induce late flowering, which can be rescued by overexpression of AIP2 (*35Sp:ABI3 35Sp:AIP2*; Zhang *et al.*, 2005). Low ABA levels throughout vegetative development may also allow a good rescue of FUS3 and ABI3 overexpression by AIP2.

### Role of AIP2 in cotyledon development

Mutations of cysteine residues within the zinc finger motif of E3s affect the interaction with E2 enzymes and thus the ubiquitination activity of E3s (Ulrich, 2002; Sadanandom *et al.*, 2012). This is also the case for AIP2, where C230S and C231S mutations abolish E3 activity of AIP2 in self-ubiquitination assays, but do not affect their interaction with the substrate (Zhang *et al.*, 2005). Seeds overexpressing RING-inactive AIP2 in the *aip2-1* background [*aip2-1 35S:AIP2*^*(C/S*)^] were previously shown to be more sensitive to ABA than *aip2-1* seeds, suggesting that AIP2 may degrade other ABA signaling factors besides ABI3 (Zhang *et al.*, 2005). Interestingly, *ML1p:HA-AIP2*^*(C/S,E/G*)^ seedlings which target the RING-inactive *AIP2*^*(C/S,E/G*)^ only to the epidermis displayed a range of developmental phenotypes that can be explained by increased activity of FUS3, ABI3, and probably other proteins. Glabrous and cotyledon-like leaves, arrested seedling growth, and delayed growth and flowering phenotypes shown by *ML1p:HA-AIP2*^*(C/S,E/G*)^ are also induced by FUS3 overexpression, and late flowering is also induced by ABI3 overexpression (Gazzarrini *et al.*, 2004; Zhang *et al.*, 2005). The inactive AIP2^(C/S,E/G)^ could act in a dominant-negative fashion, by binding to its substrates and preventing their ubiquitination by endogenous AIP2 and possibly other E3s. AIP2^(C/S,E/G)^ could also bind to functional AIP2, causing its inactivation. Combined, these would result in accumulation of AIP2 targets such as FUS3, ABI3, and other substrates in the protoderm and L1 layer, thus uncovering phenotypes not seen in *aip2-1*.

*ML1p:HA-AIP2* ^*(C/S,E/G*)^ also caused alterations in the number and morphology of the cotyledons. These defects have been previously described in mutants with altered auxin transport, synthesis, or signaling, suggesting that AIP2 may degrade proteins involved in these processes and act redundantly with other negative regulators (Bowman and Floyd, 2008; Chandler, 2008). Interestingly, auxin induces the promoters of *FUS3* and *ABI3*, both of which have been shown to regulate the expression of auxin signaling or synthesis genes positively, establishing a positive feedback regulation (Suzuki *et al.*, 2001; Gazzarrini *et al.*, 2004; Brady *et al.*, 2003; Wang and Perry, 2012; Liu *et al.*, 2013). Therefore, it is possible that AIP2^(C/S,E/G)^ alters auxin-dependent cotyledon development by interfering with the levels of FUS3 and ABI3 during embryogenesis (Fig. 7B). Interestingly, overexpression of the SnRK1 kinase AKIN10, a positive regulator of FUS3, causes similar alterations in cotyledon development, which can be partially rescued by *fus3-3* (Tsai and Gazzarrini, 2012*a*). This suggests that FUS3, or FUS3 targets, plays a role in cotyledon morphology and number. LEC2, belonging to the same B3 domain subfamily as FUS3 and ABI3, also regulates auxin synthesis and signaling (Stone *et al.*, 2008). LEC2 may also be a substrate of AIP2 since it also has B2 and B3 domains. Interestingly, loss-of-function mutations in *VAL* (*VP1/ABI3-LIKE*) genes, encoding B3 domain proteins with an EAR repression motif, result in de-repression of *LEC1* and *ABI3/FUS3/LEC2* genes, and display an increased number of cotyledons, and glabrous and cotyledon-like leaves (Suzuki *et al.*, 2007; Tsukagoshi *et al.*, 2007). Further investigation, including the identification of further AIP2 substrates, is required to understand the role of AIP2 in cotyledon development.

Studies in Arabidopsis, rice, and wheat have shown that reduced AIP2 levels result in slower germination and flowering under control conditions, but better germination and growth under salt stress (Zhang *et al.*, 2005; Park *et al.*, 2010; this study). In contrast, increased AIP2 levels induce faster germination and correlate with cultivars showing pre-harvest sprouting (PHS), which dramatically reduces crop yield and quality worldwide (Zhang *et al.*, 2005; Park *et al.*, 2010; Gao *et al.*, 2014). These findings suggest that AIP2 may control the levels of important growth regulators under stress. In the future, a better understanding of AIP2 regulation under different growth conditions and the identification of additional AIP2 substrates are needed to better understand its role in plant development and stress responses.

## Supplementary Material

Supplementary Data
