# Supplementary material for: The E3 ligase ABI3-INTERACTING PROTEIN2 negatively regulates FUSCA3 and plays a role in cotyledon development in Arabidopsis thaliana
Source: J Exp Bot. 2017 Mar 28;68(7):1555–67. doi: 10.1093/jxb/erx046 (PMC5441903; doi:10.1093/jxb/erx046)
Supplement: Supplementary Data [file erx046_Supplementary_Data.zip › supplementary_tables_S1_S2_Figures_S1_S4.pdf]

Table S1. Primers used to clone the different constructs

Construct and primers names	Primer Sequence (5' → 3')
**AIP2 (C/S, E/G)**	
AIP2(C/S)F	GTTTGGAGCAGAGGCTGAATCTTCCATCTGCAAGGAG
AIP2(C/S)R	CTCCTTGCAGATGGAAGATTCAGCCTCTGCTCCAAAC
**AD-AIP2 and AD-AIP2(C/S) in pJG4-5**	
AIP2-EcoRI-F	AA***GAATTC***ATGGATGCATCGTCTTCACCGTCTC
AIP2-XhoI-R	AA***CTCGAG***TTAAACGTACATATATTCACCTCCGC
**FLAG-AIP2-6xHis and FLAG-AIP2(C/S, E/G)-6xHis in pET28**	
FLAG-AIP2-EcoRI-F	AA***GAATTC*****ATGGATTACAAGGATGATGATGATAAG**ATGGATGCATC GTCTTCACCGTCTCCTTCCG
AIP2-XhoI-R2	AAA***CTCGAG***AACGTACATATATTCACCTCCGC
FUS5'-940-SmaI-F	GA G***CCCGGG***AA TGA TGG TTG ATG AAA ATG TGG
**ML1p:AIP2-HA and ML1p:AIP2(C/S)-HA in pEGAD**	
AIP2-3xHA Fwd	AAAAACCGGTATGTACCCATACGATGTTCCAGATTACGCTTACCCAT ACGATGTTCCAGATTACGCTTACCCATACGATGTTCCAGATTACGCT ATCGATATGGATGCATCGTCTTCACCGTC
AIP2-3xHA Rev	AAAGAATTCTTAGCAGCCGGATCTGTGG TG
ML1p Fwd	AAGGCCTTCATTTACACATCCTG
ML1p Rev	ATAACCGGTGGATTCAGGGAG

**Genotypes**	**# seeds plated**	**# arrested seedlings**
WT	100	0
	100	0
ML1:AIP2 #5	100	7
	100	5
ML1:AIP2 #10	100	4
	100	3
ML1:AIP2 #15	100	1
	100	12
ML1:AIP2 (C/S, E/G) #6	100	24
	100	22
ML1:AIP2 (C/S, E/G) #11	100	23
	100	16
ML1:AIP2 (C/S, E/G) #13	100	37
	100	40

Table S2. *ML1:-HA-AIP2* and *ML1:HA-AIP2* $^{(E/G,C/S)}$ seedling phenotypes

Number of *ML1:HA-AIP2* and *ML1:HA-AIP2* $^{(E/G,C/S)}$ seedlings showing arrested phenotypes. Seeds were sterilized, chilled for 3 days and plated on MS media under constant light at 21°C as previously described (Tsai and Gazzarrini, 2012). Two replicates are shown.

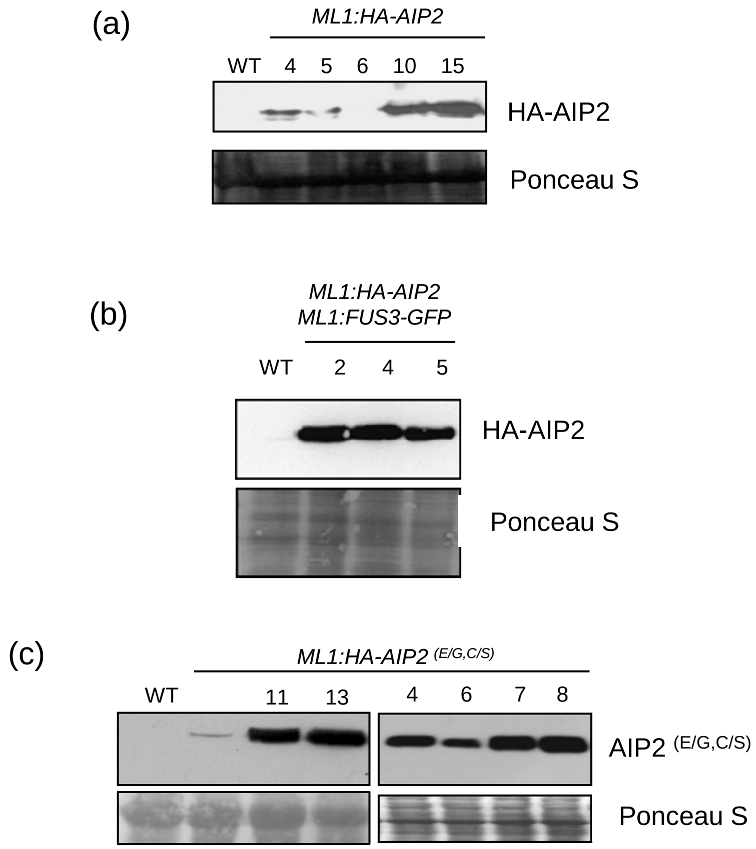

Figure S1. AIP2 protein levels in various genotypes

Western blots showing HA-AIP2 (~42 kDa) levels in 4-day-old seedlings of homozygous (A) *ML1:HA-AIP2*, (B) *ML1p:HA-AIP2 ML1p:FUS3-GFP* and (C) *ML1p:HA-AIP2* $^{(E/G,C/S)}$ seedlings. (B) *ML1p:HA-AIP2* was directly transformed into *ML1:FUS3-GFP* plants previously described (Lu et al., 2010), and independently transformed lines were selected.
Seedlings were grown under constant light at room temperature following 3 days of stratification. WT seedlings were used as the negative control. Equal amounts of total protein extract, as measured by Bradford assay, were resolved onto a 10% polyacrylamide gel. Anti-HA antibody was used to detect AIP2. Ponceau S staining is shown as the loading control.

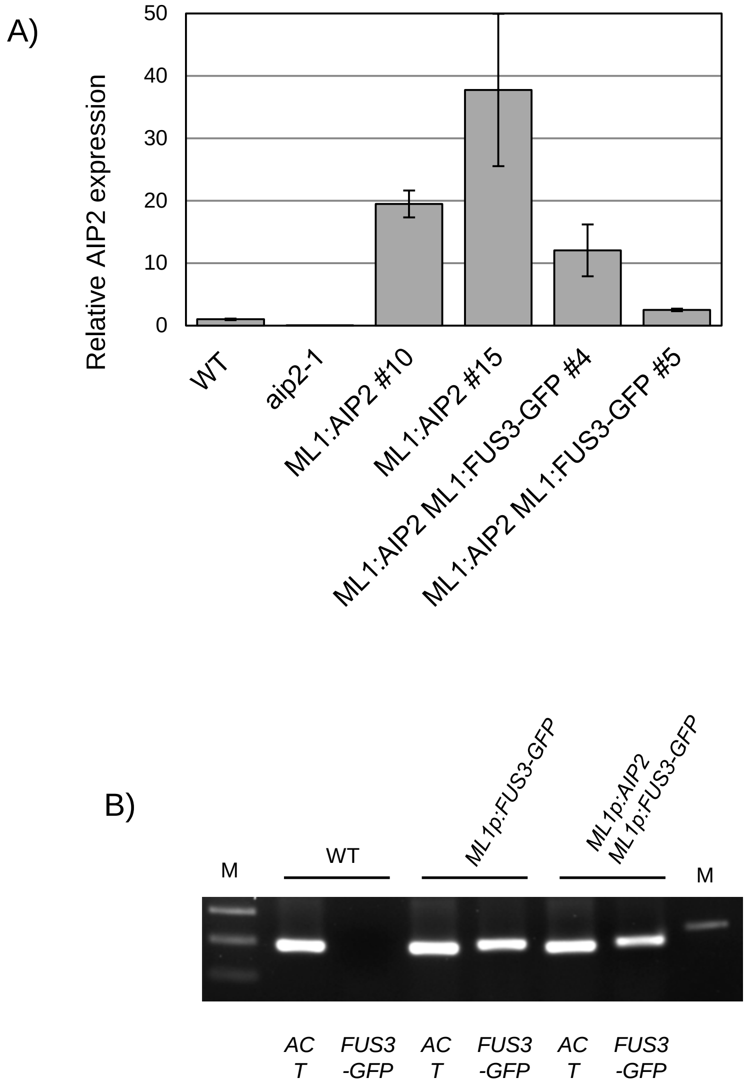

Figure S2. *AIP2* and *FUS3* transcript levels in various genotypes

A) Relative *AIP2* expression quantified by qRT-PCR. Primers spanning exons 4 and 5 of *AIP2* were used. *AIP2* expression levels were normalized to *ACTIN7*. Results from triplicate samples are shown and normalized against WT levels. Error bars indicate standard deviation. B) RT-PCR showing transcript levels of *FUS3-GFP and ACTIN7 (ACT)* in WT, *ML1:FUS3-GFP* and *ML1:FUS3-GFP ML1:AIP2* seedlings.

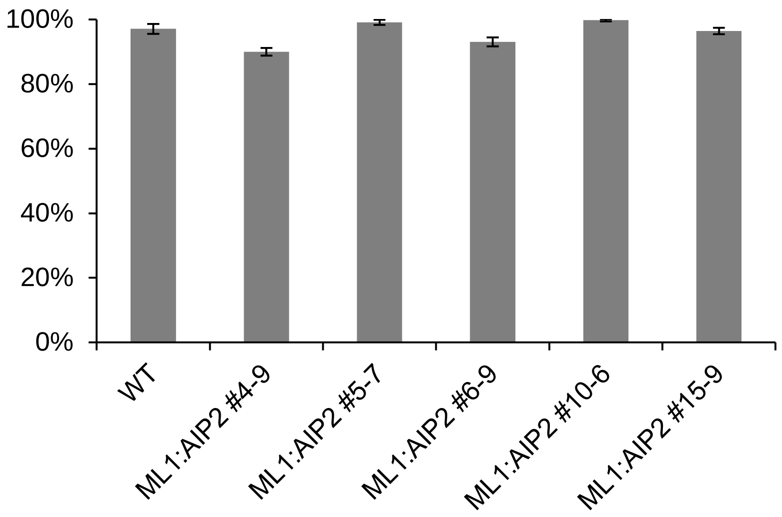

Figure S3. Germination rates of *ML1:HA-AIP2* seeds
Germination rates of dry seeds from various WT and various *ML1:HA-AIP2* lines are shown. Seeds were sterilized and plated on MS media under constant light at 21°C as previously described (Tsai and Gazzarrini, 2012). Averages from three plates with 50 seeds each + SD are shown. Similar results were obtained in different biological replicates.

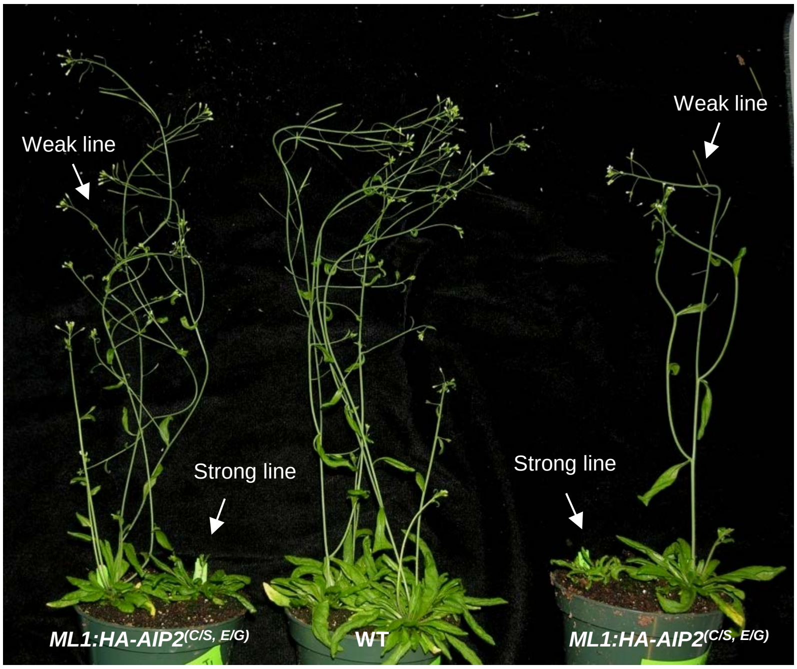

Figure S4: **Constitutive expression of RING-inactive AIP2 in the L1 layer causes delay in growth and flowering.**

Delayed growth and flowering shown by *ML1:HA-AIP2*$^{(C/S,\ E/G)}$ strong lines.
